# Southwark Guardians and "The Hospital."

**Published:** 1912-12-21

**Authors:** 


### Southwark Guardians and "The Hospital."

THE COMPLAINTS AGAINST GUY'S HOSPITAL.

At a meeting of the Southwark Board of Guardians on Thursday last Mr. W. Savage, the Vice-Chairman, who presided in the absence of the Chairman, Mr. J. O. Devereux, referred to the article in The Hospital on December 7 entitled "Bumbledom in Modern London." The article suggested that as a friendly conference between the Guardians and the Governors of Guy's Hospital had not taken place the Local Government Board, before whom the facts had been placed by the Guardians, "would no longer suffer the present scandal to endure in Southwark without the institution of an immediate and thorough inquiry."

Mr. Savage said that the article was based on an inaccurate conception of the facts. What the Southwark Board of Guardians objected to was that the authorities at Guy's Hospital were in the habit of sending to the relief station for transmission to the Southwark Infirmary patients who did not reside in the parish and had no claim whatever, except those of a common humanity, upon the ratepayers of Southwark. Many of these came to the hospital from long distances; they had been out-patients at the hospital for some months, and when they became too ill for the hospital authorities to deal with them they were simply drafted on to the Southwark Poor-Law authority. This fact was borne out by figures. In twelve months out of 162 patients treated in Southwark institutions, referred to them by the authorities at the hospital, only 71 belonged to Southwark. Of the remainder 45 were homeless and 46 belonged to other parishes. Why, he asked, should Southwark be called upon to provide a special fully-equipped infirmary at the doors of Guy's Hospital to deal with 91 persons in twelve months whom the hospital authorities declined to find beds for? In no other parish in London did such an unsatisfactory state of affairs exist as in Southwark, simply because the hospital authorities in other centres treated all their patients with humanity and kindness, and endeavoured as far as possible to save them from the Poor-Law taint. As Mr. Devereux had remarked on a previous occasion, the authorities at Guy's Hospital appeared to be only too anxious to pauperise the people, and the Southwark Board of Guardians was not out to assist them in their efforts.

It was evident that these facts, continued Mr. Savage, were not known to the Editor of The Hospital, nor that on several occasions deputations from the Guardians had waited upon the authorities at Guy's Hospital urging them to adopt a course that would inflict less suffering on the poor, not merely the sick poor of Southwark, as stated in The Hospital, but the sick poor of London and those who came for treatment to the hospital from outside the London area. The Guardians were called upon, at the instigation of the authorities of the hospital, to provide beds for every patient in a dying or moribund condition wherever they came from, and he felt sure that neither the Local Government Board nor Mr. John Burns would uphold for a single instant the demand of the hospital authorities. There had been no complaints from the Southwark poor as to the situation of their infirmary, and for the first time he learnt in the columns of The Hospital that the Guardians had failed to provide necessary ambulance and hospital accommodation in the heart of the district.

If it was no part of the duty or work of Guy's Hospital to undertake the hospital service imposed by law upon the Guardians, as The Hospital stated, said Mr. Savage, neither was it the duty of the Guardians to provide infirmary accommodation for 91 persons sent to them in one year by the authorities at the hospital, none of whom belonged to Southwark. At the same time the Guardians provided beds for these strangers, even though their own infirmary was so crowded that they had to place beds for them on the floor. He felt sure that if the authorities at the hospital were actuated with a desire to relieve suffering they would have adopted the same course and not have sent the patients away to die a few hours or days later. He had been present on several deputations at Guy's Hospital on this matter and, although for a short time after representations had been made to the authorities there was an alteration for the better, after a little while the same unsatisfactory results took place. The Guardians had only swept aside the suggestion for another friendly conference after they had found that the previous conferences were futile. The authorities at Guy's Hospital declined at times to admit patients suffering from injuries received in street accidents, sending them on to the infirmary to be treated. If this was not manufacturing paupers, he failed to know what was.

Mr. Savage then went on to refer to a few typical cases sent to the Southwark Infirmary from Guy's Hospital. In one case a man suffering from epithelioma of the neck had been an out-patient at the hospital. He was found at 9.30 in the morning in the Borough Market in a state of collapse by the police and was taken to the hospital, where he was kept for two hours. At the end of that time he was recommended for admission to the infirmary by the hospital doctor. On arrival at the relief station he was in a state of collapse, and even the police expressed their surprise at the action of the hospital authorities. As soon as he came under the notice of the Poor-Law authorities the man became no longer a patient, but "a pauper." The man was so ill that he had to be sent to St. George's Workhouse. In another case a woman unconscious for fourteen hours after a fall was sent from the hospital to the infirmary, where she died within a few hours. The house surgeon at the hospital said there appeared to be not much the matter with her. These cases could be multiplied without number, added Mr. Savage. The Guardians had no quarrel with Guy's Hospital, their sole desire was to alleviate the sufferings of the sick poor by removing the present unsatisfactory method of sending dying patients from the hospital to the infirmary. The Guardians welcomed a full inquiry, as, in the words of The Hospital, "it was intolerable that modern London should be longer exhibited to the world, through the fault of those responsible, as tolerant" of these methods.

The Board concurred in the views expressed by Mr. Savage.

[We are glad to publish Mr. W. Savage's speech, and the contradictory views which have been expressed by both parties to the discussion serve only to illustrate our reiterated conviction that the matters in dispute are of a nature which can be dealt with on a satisfactory basis only by a Government inquiry conducted through the Local Government Board.—Ed. The Hospital.]

